# Proteomic Reveals Reasons for Acquired Drug Resistance in Lung Cancer Derived Brain Metastasis Based on a Newly Established Multi-Organ Microfluidic Chip Model

**DOI:** 10.3389/fbioe.2020.612091

**Published:** 2020-12-22

**Authors:** Mingxin Xu, Yingyan Wang, Wenzhe Duan, Shengkai Xia, Song Wei, Wenwen Liu, Qi Wang

**Affiliations:** ^1^Department of Respiratory Medicine, The Second Hospital, Dalian Medical University, Dalian, China; ^2^Laboratory Center for Diagnostics, Dalian Medical University, Dalian, China; ^3^Cancer Translational Medicine Research Center, The Second Hospital, Dalian Medical University, Dalian, China

**Keywords:** lung cancer, brain metastasis, microfluidic organ-on-a-chip, drug resistance, proteomic

## Abstract

Anti-tumor drugs can effectively shrink the lesions of primary lung cancer; however, it has limited therapeutic effect on patients with brain metastasis (BM). A BM preclinical model based on a multi-organ microfluidic chip has been established proficiently in our previous work. In this study, the BM subpopulation (PC9-Br) derived from the parental PC9 cell line was isolated from the chip model and found to develop obvious resistance to antineoplastic drugs including chemotherapeutic agents (cisplatin, carboplatin, pemetrexed) and tyrosine kinase inhibitors (TKIs) which target epidermal growth factor receptor (EGFR); this suggested that the acquisition of drug-resistance by brain metastatic cells was attributable to the intrinsic changes in PC9-Br. Hence, we performed proteomic and revealed a greatly altered spectrum of BM protein expression compared with primary lung cancer cells. We identified the hyperactive glutathione (GSH) metabolism pathway with the overexpression of various GSH metabolism-related enzymes (GPX4, RRM2, GCLC, GPX1, GSTM4, GSTM1). Aldehyde dehydrogenases (ALDH1A1, ALDH3A1) were also found to be upregulated in BM. What's more, loss of EGFR and phosphorylated EGFR in PC9-Br gave reasons for the TKIs resistance. Collectively, our findings indicated potential mechanisms for the acquirement of drug resistance occurred in BM, providing new strategies to overcome therapeutic resistance in lung cancer BM.

## Introduction

Lung cancer has been the most common cause of cancer death worldwide with high morbidity and mortality (Siegel et al., 2019). Non-small cell lung cancer (NSCLC), as the most common pathological type of lung cancer with the proportion of 85%, is characterized by a high incidence of distant metastasis within which brain metastasis (BM) accounts for 40% (Steeg et al., 2011; Ali et al., 2013). The prognosis of BM patients is extremely poor with a median survival time of only 4–6 months due to limited treatment alternations (Cheng and Perez-Soler, 2018). Currently available therapeutic approaches for BM include a combination of surgery, radiotherapy, chemotherapy, molecular targeted therapy, or anti-angiogenesis therapy, as applicable; however, the therapeutic efficacy is particularly poor (Shi et al., 2017).

Drug treatments which mainly based on chemotherapy is an indispensable treatment for BM patients since there are very limited therapeutic indications for surgery and radiation therapy (Yousefi et al., 2017; Achrol et al., 2019), however, the drug response rate in BM populations is extremely low (Sandler et al., 2000; Schiller et al., 2002; Barlesi et al., 2011; Bailon et al., 2012). It is believed that there are two main factors which contribute to the poor efficacy of drug therapy against BM: the presence of blood-brain barrier (BBB) and tumor-intrinsic changes induced by metastasis events. BBB provides a sanctuary site for tumor to escape drug treatment as the barrier can significantly prevent the penetration of anti-tumor drugs into both tumor and brain tissues. However, recent studies have proposed that the disruption of BBB during BM enables the invasion and colonization of tumor cells in brain parenchyma (Li et al., 2012; Liu et al., 2019), suggesting that the barrier which hinder the entry of drugs was impaired during BM. This holds the point that endogenous alteration of metastatic cells may play a vital role in the acquisition of drug resistance. However, it remains unknown to a large extent.

Traditional research models of tumor brain metastasis were established by injecting tumor cells into the left cardiac ventricle of athymic nude mice. Brain metastasis cells (BrMs) derived from parental tumor cells were subsequently isolated from extracted brain metastases (Bos et al., 2009; Liu et al., 2019; Shah et al., 2020). Although BrMs were proved to possess enriched BM properties, the cells did not undergo the whole BM pathological process since the progression of tumor cells *in situ* was skipped. It has to be admitted that intracardiac injection is a better choice to initiate BM efficiently than orthotopic injection. Under the condition of orthotopic injection, animals often die before brain metastasis occurs since they cannot bear the tumor load *in situ*. Hence, there is an urgent need for a reliable and efficient model which can faithfully mimic the entire BM process. Recently, microfluidic organ-on-a-chips have been developed and applied as powerful tools for medical research, enabling the creation of more *in vivo*-like *in vitro* models with high throughput (Ashammakhi et al., 2020; Ding et al., 2020; Moradi et al., 2020; Steinway et al., 2020). In our previous work, a multi-organ microfluidic chip, which consists of two organ chip units–an upstream “lung” and a downstream “brain” unit, was established and proved to be able to effectively reproduce the whole pathological process of lung cancer BM, providing a novel model for BM research (Liu et al., 2019).

In this study, we isolated the brain metastatic cells (PC9-Br) from the metastases on the BM chip and found that PC9-Br developed a significant resistance to multiple anti-tumor drugs compared with its parental PC9 cells. Further proteomic was carried out to reveal possible reasons for the acquirement of drug resistance in the BM, followed by the selection of differentially expressed proteins by western blotting validation. We found the glutathione (GSH) metabolism pathway, which plays an important role in intracellular antioxidant stress response, was remarkably hyperactive in BM, along with the overexpression of a series of GSH metabolism-related enzyme (GPX4, RRM2, GCLC, GPX1, GSTM4, GSTM1). Aldehyde dehydrogenases (ALDH1A1, ALDH3A1), which have been reported to mediate the acquired drug resistance of tumor cells, were also found to be upregulated in BM. What's more, reduced expression of epidermal growth factor receptor (EGFR) and phosphorylated EGFR gave a reasonable explanation for the EGFR targeted drugs resistance of PC9-Br compared with PC-9 parental cells.

## Materials and Methods

### Cell Culture and Drugs

The human lung cancer cell line PC9 was used as the primary lung cancer model and parental cells of BM derivative (PC9-Br) on the chip. To establish the bionic microenvironment for primary lung cancers and secondary BM of lung cancers, human bronchial epithelial cells (16HBE), human pulmonary microvascular endothelial cells (hPMEC), human lung fibroblasts (HFL1), and human mononuclear cells (THP-1) were co-cultured in the upstream “lung” unit while human astrocytes (HA-1800) and human brain microvascular endothelial cells (hBMVEC) were co-cultured dynamically in the downstream “brain” unit as previously reported (Xu et al., 2016; Liu et al., 2019). The purchase sources and culture conditions of all cell lines have also been- described in detail previously (Liu et al., 2019).

Cisplatin (MB1055), Carboplatin (MB1297), Paclitaxel (MB1178), Pemetrexed Disodium (MB1183), Etoposide (MB1102), Gefitinib (MB1112) and AZD3759 (MB4776) were purchased from Meilunbio (China).

### Construction and Operation of the Multi-Organ Microfluidic Chip Model to Mimic the Pathological Process From Primary Lung Cancer to Brain Metastasis

It has been described in detail in our previous work (Xu et al., 2016; Liu et al., 2019). In short, after the establishment of upstream bionic “lung” and downstream bionic “brain,” human lung cancer cells PC9 which stably express green fluorescent protein (GFP)-luciferase fusion protein, were introduced into the upstream unit to form primary lung cancer model. After the growth, transition and invasion on the upper stream “lung,” primary PC9 cells invaded the circulation and were transported to the downstream “brain” along with the dynamic fluid medium. Then metastatic cells attached to and trans-migrate through BBB structure, colonized in brain parenchyma and finally formed brain metastases. The entire BM process on the chip was dynamically monitored with an Olympus IX81 fluorescence microscope (Olympus Corporation, Japan).

### Extraction and Isolation of Brain Metastatic Cells of Parental Lung Cancer Cells PC9 (PC9-Br) From Chips

Dovetail clips were used to completely block the connecting passage between upstream and downstream unit. A syringe pump (Pump 11 Elite Series Pumps, USA) was connected to the injection port of brain parenchymal chamber to drive the flow of 0.25% trypsin-EDTA solution (Gibco, Invitrogen, Inc, USA) at the speed of 0.1 μl/min, while a sterile collection tube was connected to the outlet of brain parenchymal chamber to collect the mixed cell suspensions of brain metastases. After collection, complete medium was added into the tube to stop digestion. A mixed mass of various cells was obtained through centrifuge. Then the BM derivative cells (PC9-Br) of parental PC9 were isolated by GFP fluorescence sorting with a flow cytometer (FACSAriaTMII, BD, USA) and transferred to cell culture dish for routine culture. The sorting efficiency was verified with a fluorescence microscope.

### Invasion Assay

Twenty-five thousand cells in 200 μl serum-free medium were seeded into matrigel-coated top chamber of 24-well Transwell inserts (#3422, Corning, USA) while the bottom chambers were filled with 500 μl complete medium. After 24 h the non-invading cells on the upper side were removed with cotton swabs and the invaded cells on the lower surface of the insert were fixed with 4% paraformaldehyde and stained with 0.5% crystal violet (KeyGEN BioTECH Corp., Ltd, China). Images were captured with a microscope (Leica, TCSSP5II). The invaded cells in five random fields of one image were counted and the invasion ability was standardized by making a ratio of PC9-Br invaded cell number to the parental PC9 invaded cell number (100%).

### Trans-Endothelial Assay

Twenty thousand were seeded and cultured on the fibronectin-coated 24-well Transwell inserts (#3422, Corning, USA) for 72 h and allowed to form an integrated endothelial mono-layer. Then 25,000 cancer cells in 200 μl serum-free medium were seeded on the insert above the endothelial monolayer while 250 μl complete medium was added to the lower side. After 36 h of culture, the cells on the upper side were removed with cotton swabs and the trans-migrated cells on the lower surface of the insert were fixed with 4% paraformaldehyde and stained with crystal violet (KeyGEN BioTECH Corp., Ltd, China). Images were captured with a microscope (Leica, TCSSP5II). The trans-migrated cells in five random fields of one image were counted and trans-endothelial ability was standardized by making a ratio of PC9-Br trans-migrated cell number to the parental PC9 trans-migrated cell number (100%).

### Animal Study

The animal study was reviewed and approved by the Animal Ethics Review Committee of Dalian Medical University (No.00122773). Female BALB-c-nu mice (4–6 weeks) were obtained from the Beijing Vital River Laboratory Animal Technology Co. Ltd., China.

Each mouse was anesthetized with ketamine (100 mg/kg body weight; Sigma, USA) and xylazine (10 mg/kg body weight; Sigma, USA) and then inoculated with 1,000,000 PC9 or PC9-Br cells in 100 μl PBS by intracardiac injection. The BM events in mice were recognized by bioluminescence imaging (BLI) with an IVIS Spectrum Xenogen machine (PerkinElmer, USA) as previously described (Xu et al., 2016; Liu et al., 2019). In brief, after mice were anesthetized and injected retro-orbitally with D-Luciferin (150 mg/kg body weight; Promega, USA), images were obtained and analyzed with Living Image software (version 2.50).

### Cell Viability Assay

Cell Counting Kit-8 (CCK8, K1018, ApexBio, USA) was used to assess cell viability according the manufacturer's instructions. Briefly, 5,000 cells were seeded to each well of 96-well plates and allowed to adhere to the wall. Then the culture medium was removed and cells were treated with specific drugs for 72 h. CCK8 solution was added to each well and incubated for 2 h at 37°C in the dark following the instructions. Color change was measured at 450 nm with a microtiter plate reader, and the OD value was observed to be directly proportional to cell viability.

### Quantitative Tandem Mass Tag (TMT)-Based Proteomics

This work was supported by the Jingjie PTM BioLab (Hangzhou, China) Co. Ltd. Main experimental procedures of TMT proteomics analysis, including protein extraction, trypsin digestion, TMT labeling, HPLC fractionation, LC-MS/MS analysis, database search and bioinformatics analysis, are presented in the Supplementary methods in detail.

### Western Blot

RIPA cell lysis buffer containing a protease inhibitor cocktail (Meilunbio, China) and a phosphatase inhibitor cocktail (Sigma, USA) was used to dissolve proteins extracted from cells. The BCA assay kit (Thermo Fisher Scientific Inc., USA) was used to measure the protein concentration. Protein lysates were then separated by sodium dodecyl sulfate-polyacrylamide gel electrophoresis (SDS-PAGE) and transferred onto nitrocellulose membranes (Millipore, Billerica, USA). The membranes were blocked in 5% skimmed milk solution in 0.05% Tris-buffered saline/Tween-20 (TBST) and then incubated with primary antibodies against GPX4 (1:1,000 dilution; ab125066, Abcam, UK), RRM2 (1:1,000 dilution; ab57653, Abcam, UK), GCLC (1:1,000 dilution; ab190685, Abcam, UK), GPX1 (1:1,000 dilution; ab108427, Abcam, UK), GSTM4 (1:1,000 dilution; ab233281, Abcam, UK), GSTM1 (1:1,000 dilution; ab113432, Abcam, UK), ALDH3A1 (1:1,000 dilution; ab129022, Abcam, UK), ALDH1A1 (1:1,000 dilution; ab52492, Abcam, UK), EGFR (1:1,000 dilution; SB52894, Abcam, UK), p-EGFR (1:1,000 dilution; ab32430, Abcam, UK), phospho-NF-κB p65 (Ser536,1:1000 dilution; #3033, Cell signaling technology, USA) and NF-κB p65 (1:1000 dilution; #8242, Cell signaling technology, USA). After washing with 0.05% TBST, the corresponding secondary antibodies conjugated with horseradish peroxidase (1:5,000; Proteintech, China) were further used. The ECL western blotting substrate (Tanon, China) was used to analyze the chemiluminescence of the blots. Protein expression was quantified by Image J software (National Institutes of Health, USA). The tests were performed with triplicated samples to diminish variability.

### Statistical Analysis

Statistical analysis was performed using GraphPad Prism software 5.0 and SPSS 16.0 statistical software package. Quantitative data are presented as mean (± standard deviation) values from at least 3 independent experiments. Differences between two groups were assessed using the *t*-test.

## Results and Discussion

### The Brain Metastatic Derivative Isolated From BM Chip Possessed Enriched BM Activities Compared to Parental Cells *in vitro* and *in vivo*

**Figure 1:**
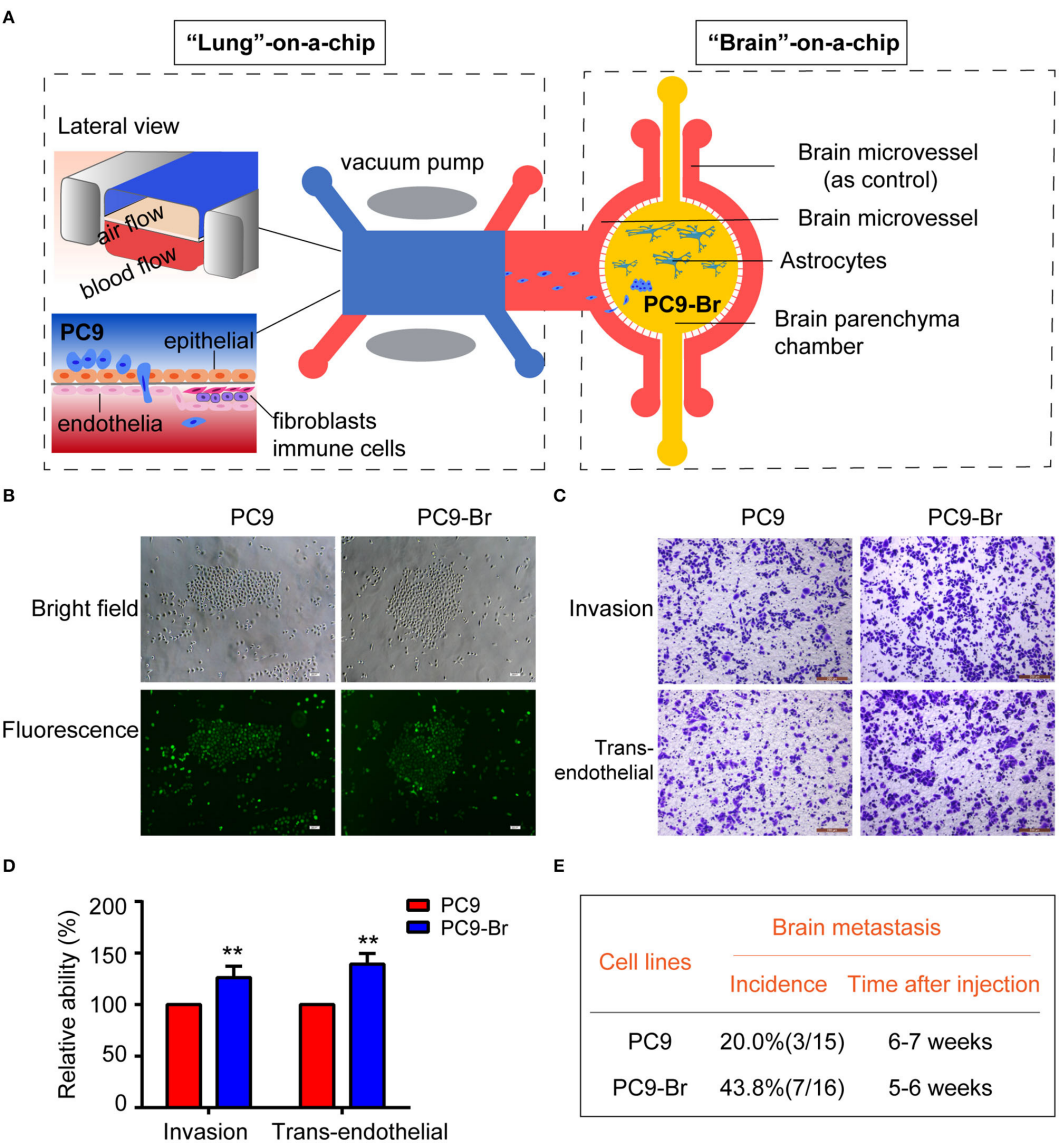
The brain metastatic derivative isolated from BM chip possessed enriched BM activities compared to parental cells *in vitro* and *in vivo*. **(A)** Schematic illustration of the construction and operation of BM process on the multi-organ microfluidic chip as previously described (Liu et al., 2019). **(B)** Representative co-images of PC9 and PC9-Br cells under the bright field and fluorescence. Scar bar, 200 μm. **(C,D)** Representative images showing the invasion and trans-endothelial migration of the PC9 and PC9-Br cells on the Transwell (scale bar, 200 μm). *n* = 3, **p < 0.01. **(E)** Frequency and time circle of BM after inoculation of PC9 or PC9-Br cells in nude mice.

The whole pathological process of BM was simulated on the established multi-organ microfluidic chip by introducing the parental lung cancer cells PC9 into the upstream bionic lung unit as previously reported (Liu et al., 2019), as illustrated in Figure 1A. The brain metastases formed in the downstream bionic brain unit were harvest and the brain metastatic populations (PC9-Br) were isolated by GFP fluorescence sorting. The sorting efficiency was assessed with cell co-imaging under the bright field and fluorescence (Figure 1B), ensuring that the isolated cells were GFP-expressed tumor cells rather than non-fluorescent mesenchymal cells. Then the BM activities of PC9-Br cells and corresponding parental cells were evaluated *in vitro* and *in vivo*. Figures 1C,D showed that PC9-Br cells exhibited stronger invasion and trans-endothelial abilities than PC9 cells. For *in vivo* experiments (Figure 1E, Supplementary Figure 1), the PC9-Br cells formed BM in 43.8% of mice with a 5–6 weeks tumor formation cycle, while the parental PC9 cells exhibited a BM efficiency of 20.0% with a 6–7 weeks tumor formation cycle. Taken together, the brain metastatic derivative isolated from BM chip possessed enriched BM activities compared to parental cells *in vitro* and *in vivo*. The results also displayed the great potential of organ-on-a-chips in constructing complicated disease models and exploring pathological mechanisms.

### The Acquirement of Obvious Resistance to Multiple Anti-tumor Drugs Was Found in PC9-Br Cells

**Figure 2:**
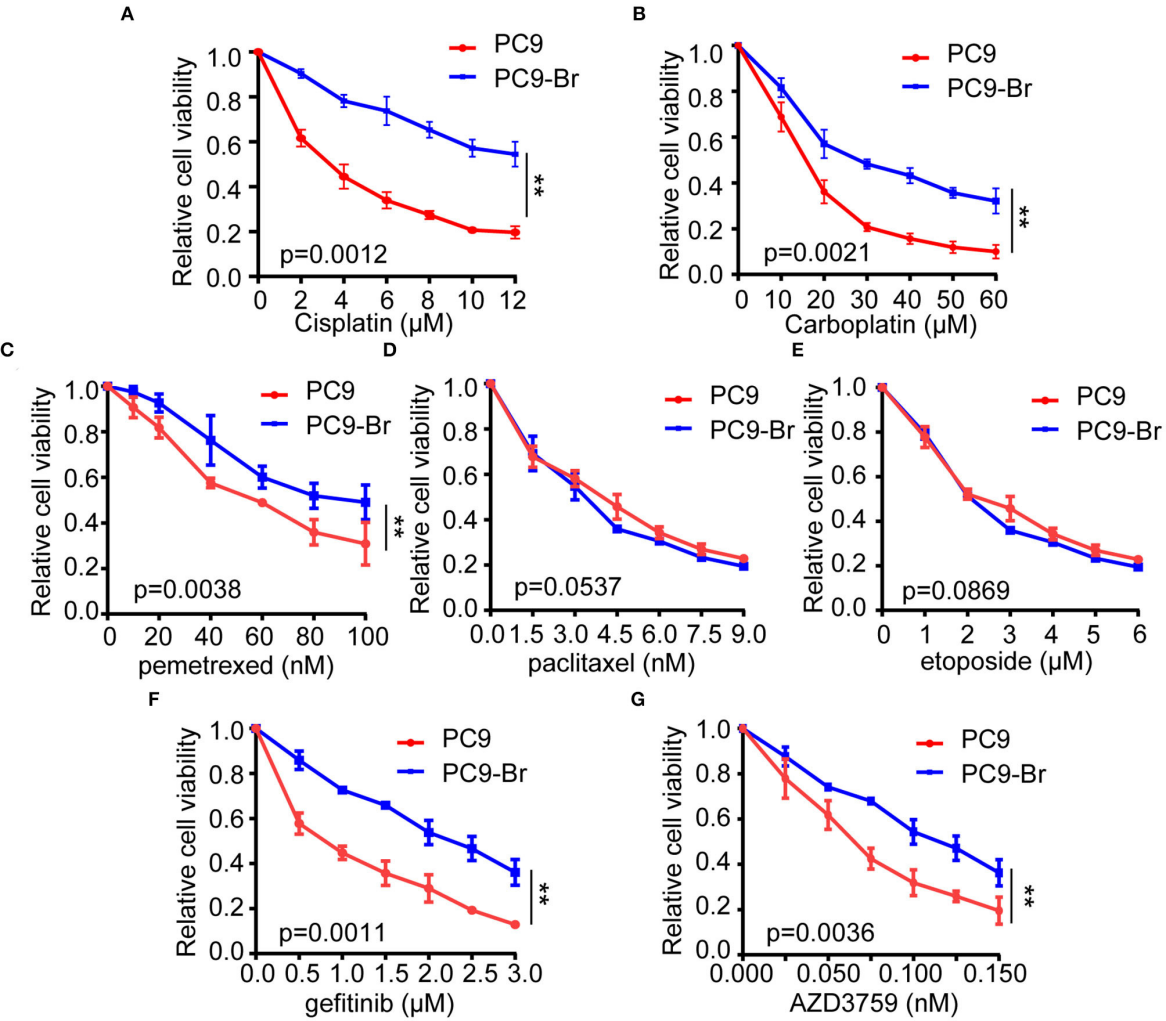
The acquirement of obvious resistance to multiple anti-tumor drugs was found in PC9-Br cells. PC9 and PC9-Br cells were treated with different doses of cisplatin **(A)**, carboplatin **(B)**, pemetrexed **(C)**, paclitaxel **(D)**, etoposide **(E)**, gefitinib **(F)**, and AZD3759 **(G)** for 72 h and CCK-8 assays were performed to determine their viability. *n* = 3, **p < 0.01.

Current drug treatments for patients with lung cancer BM mainly include chemotherapy and targeted drug therapy. Platinum compounds (cisplatin and carboplatin) and pemetrexed, alone or in combination (etoposide, paclitaxel, and radiotherapy) are the most commonly used chemotherapy regimens against BMs from NSCLC (Mehta et al., 2010; Barlesi et al., 2011; Bailon et al., 2012; Shi et al., 2017; Yousefi et al., 2017; Franchino et al., 2018). Since about 33% of patients with non-small cell lung cancer (NSCLC) and EGFR mutations develop BMs, different generations of tyrosine kinase inhibitors (TKIs) were commonly used to target BMs (Burel-Vandenbos et al., 2013; Sekine and Satoh, 2017; Zhuang et al., 2019). Gefitinib is the most typical first-generation TKI and AZD3759 is characterized by its strong ability to effectively penetrate the BBB (Tan et al., 2017; Hochmair, 2018). Historically, the limited use of anti-tumor drugs in cancer patients owing to the presence of the BBB. However, recent studies have proved that the BBB was disrupted in BMs which results in an increased exposure to systemic drugs, suggesting the sensitivity of tumor cells to drugs is the main determinant of therapeutic efficacy. Hence, we explored whether there were differences in drug sensitivity between PC9-Br and PC9 by treating these two groups of cells with the above agents. Results of cell viability assay indicated that PC9-Br developed obvious resistance to platinum compound (both to cisplatin and carboplatin, Figures 2A,B), pemetrexed (Figure 2C) and TKIs (both to gefitinib and AZD3759, Figures 2F,G) compared with the parental PC9 group. However, there was no significant difference in the response to paclitaxel and etoposide (Figures 2D,E). Similar changes of drug sensitivity were found in animal model derived BM cells PC9-BrM3 (Supplementary Figures 2A–E), a highly brain metastasis cell line by injecting parental tumor cells PC9 into the left-ventricle of immunodeficient mice and isolating the metastatic cells from harvested brain metastases three times repeatedly in our previous work (Liu et al., 2019). Collectively, these results indicated BM cells acquired obvious resistance to multiple anti-tumor drugs. In addition, these findings strongly suggested that endogenous factors of BM cells were the culprit of the poor drug efficacy.

### Proteomics Identified the Hyperactive GSH Metabolism Pathway in PC9-Br Cells

**Figure 3:**
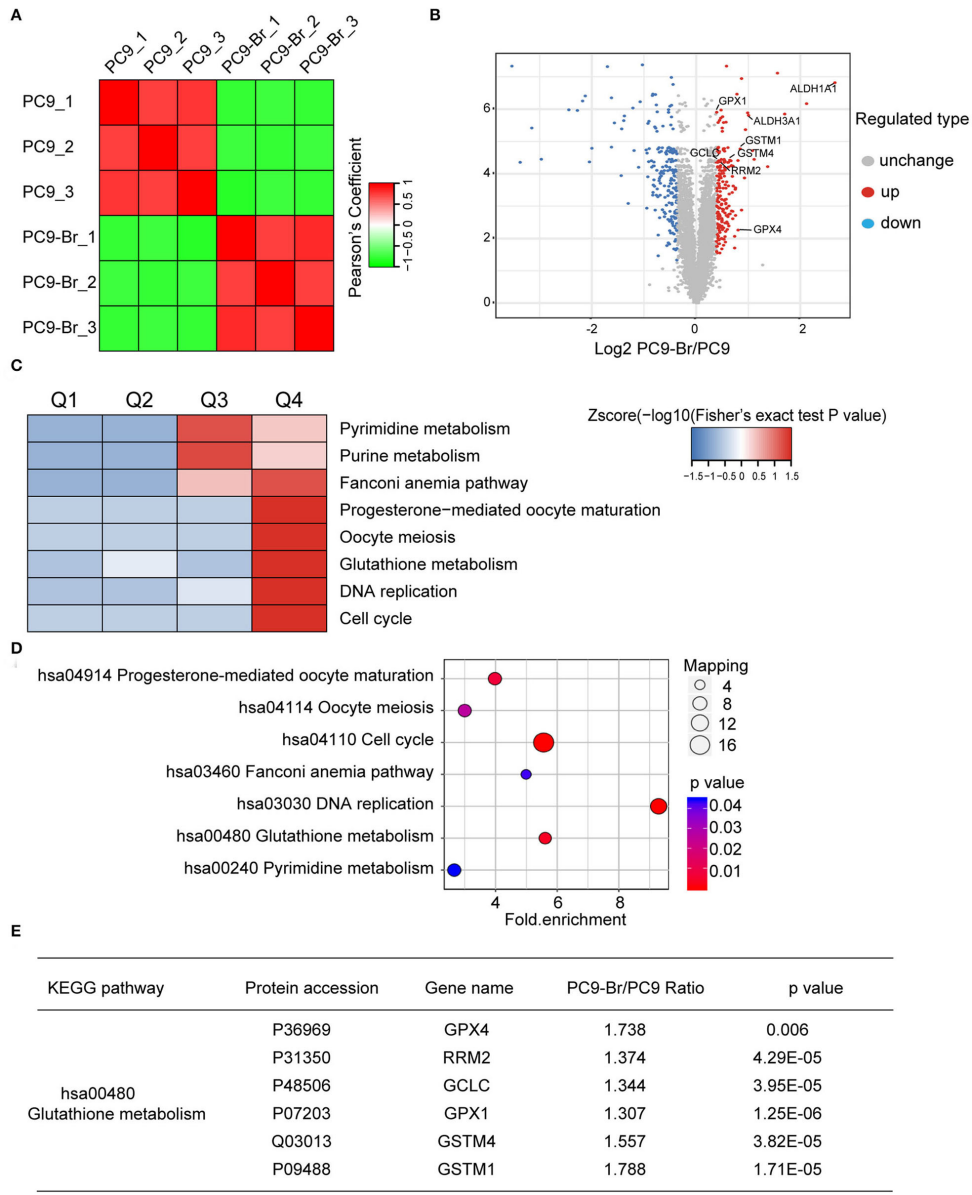
Proteomics identified the hyperactive GSH metabolism pathway in PC9-Br cells. **(A)** Correlation analysis for reproducibility of proteomics data. **(B)** Volcano plot showed the distribution of differential proteins in Q4 group (ratio fold >1.3). The horizontal axis is Log2 transformed protein expression ratio, and the vertical axis is –Log10 transformed *p-*value. Red dot: up-regulated protein; blue dot: down-regulated protein. **(C)** Functional KEGG enrichment cluster image showing the common enriched pathways in PC9-Br cells. **(D)** Results of KEGG enrichment analysis of differential proteins in groups (PC9-Br/PC9) visualized in bubble diagram (including the number of respective involved proteins and the corresponding *p*-values). **(E)** The specific differential proteins identified in groups (PC9-Br/PC9) involved in GSH metabolism pathway are listed.

Proteomics has become a powerful and promising complementary technology to provide insights into diseases at a more phenomenological level (Altelaar et al., 2013). We employed proteomics to find the potential cellular endogenous factors resulting in the acquired drug resistance of PC9-Br compared to parental PC9 cells. Figure 3A showed the good reproducibility of proteomics data. Differential proteins with different ratio folds were identified. To find the potential correlation of protein functions with differential ratio folds, we divided them into 4 parts according to their ratio folds, called Q1 to Q4: Q1 (0 < Ratio ≤ 1/1.3), Q2 (1/1.3 < Ratio ≤ 1/1.2), Q3 (1.2 < Ratio ≤ 1.3) and Q4 (Ratio >1.3). Then we performed KEGG pathway enrichment for each Q group and the results showed a number of pathways were enriched in Q4 protein group with higher ratio folds (Figure 3C). Further analysis was carried out on Q4 group while 200 upregulated proteins and 203 downregulated proteins were identified (Figure 3B). The specific enrichment results of KEGG pathways in Q4 were visualized by the number of proteins involved respectively and their corresponding p-values (Figure 3D). The cell cycle, DNA replication regulation and glutathione (GSH) metabolism pathway were identified to be enriched significantly in PC9-Br cells, giving potential explanations for the enhanced BM abilities, as well as the acquirement of multi-drugs resistance of PC9-Br cells. Hyperactive cell cycle and DNA replication regulations has been well-recognized as one of major malignant behaviors in tumors (Hanahan and Weinberg, 2011), contributing greatly to the proliferation of tumor cells, while GSH metabolism has been demonstrated as the main factor which cause multi-drug resistance, giving a more specific explanation for the drug resistance occurred in PC9-Br cells. GSH, as the most abundant antioxidant found in living organisms, plays a vital role in maintaining cellular redox homeostasis. Increasing evidence has shown that abnormal GSH metabolism is the main factor causes drug resistance by binding or reacting with drugs, interacting with reactive oxidative species (ROS), preventing protein or DNA damage, or participating in DNA repair process (Traverso et al., 2013; Bansal and Simon, 2018). Hence, we listed the differential proteins involved in GSH metabolism pathway (Figure 3E). The results showed that a series of enzymes related to GSH metabolism were significantly overexpressed, indicating that GSH metabolism is hyperactive in PC9-Br cells, which provided reasonable clues for exploring drug resistance developed in BM.

### Drug-Resistance Related Proteins Were Confirmed to Be Regulated in BM Cells

**Figure 4:**
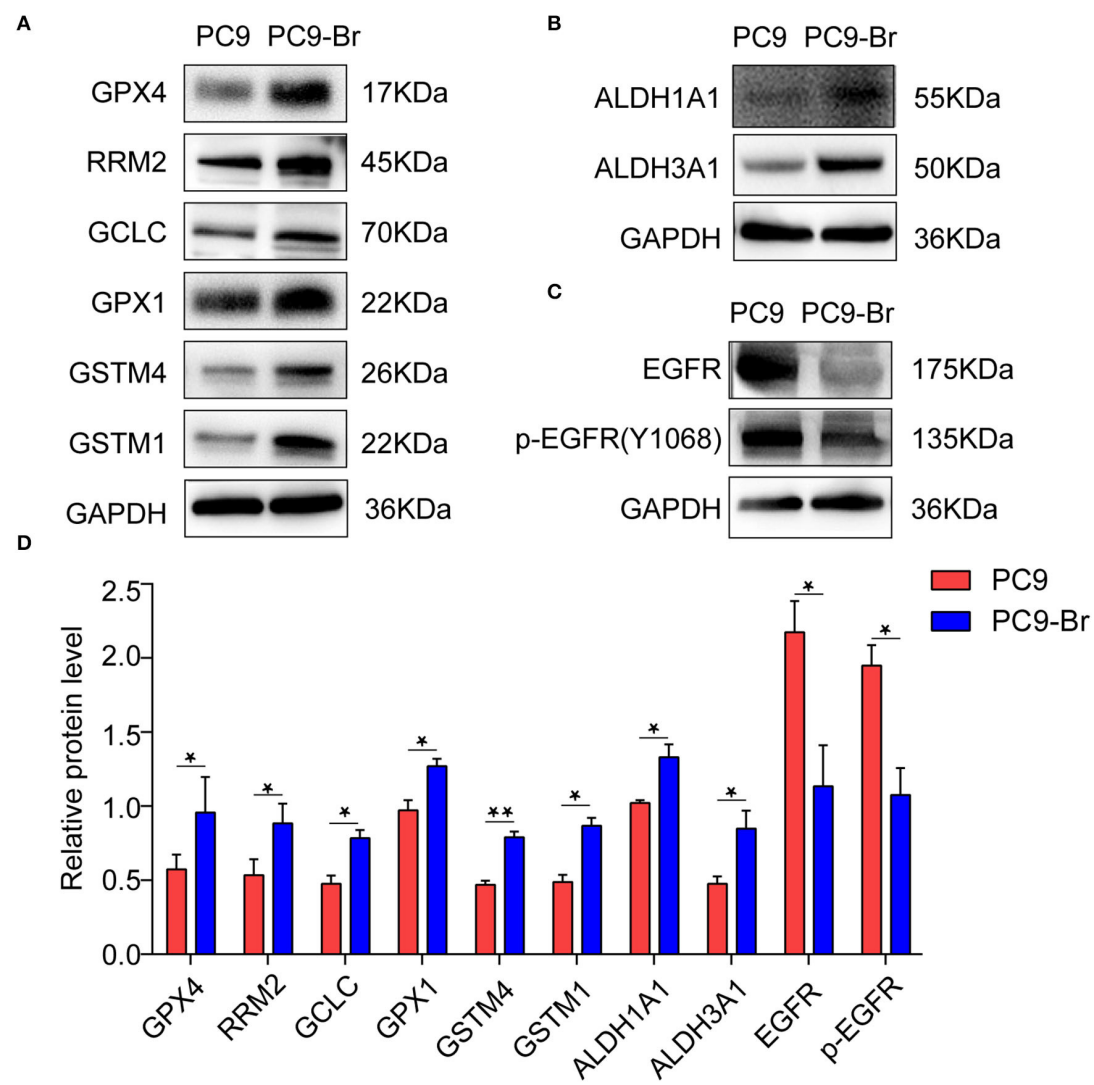
Drug-resistance related proteins were confirmed to be regulated in PC9-Br cells. Representative western blot images showing the expression of GSH metabolism related enzymes **(A)**, ALDH1A1 and ALDH3A1 **(B)**, and EGFR/p-EGFR **(C)** in PC9 and PC9-Br cells. **(D)** Quantitative results of western blotting images for proteins. *n* = 3, *p < 0.05, **p < 0.01.

Since a string of GSH metabolism related enzymes were found overexpressed in PC9-Br by proteomics, the expression of these enzymes (GPX4, RRM2, GCLC, GPX1, GSTM4, GSTM1) was further verified to be upregulated in PC9-Br by western blotting (Figures 4A,D). GSH dependent peroxidases (GPXs) catalyze the conversion of GSH to GSSG under oxidative stress. GPX1 was found to promote the resistance to cisplatin of NSCLC (Chen et al., 2019a), while GXP4 was widely studied as a novel ferroptosis regulator (Dixon et al., 2012; Yang et al., 2014). In addition, increasing evidence suggests that drug-tolerant persister tumor cells are susceptible to GPX4 inhibition (Hangauer et al., 2017). The expression and enzymatic activity of GCLC constitute rate-limiting steps for GSH synthesis (Lu, 2009, 2013), while glutathione transferases (GSTs) have been shown to be involved in the development of multi-drug resistance (MDR) toward chemotherapeutic agents (Townsend and Tew, 2003; Chatterjee and Gupta, 2018). RRM2 was also proved to mediate multi-drug resistance in multiple malignant tumors (Shah et al., 2014; Chen et al., 2019b). Furthermore, aldehyde dehydrogenases 1 and 3 (ALDH1A1 and ALDH3A1), which were well recognized to play functional roles in drug resistance of NSCLC (Rebollido-Rios et al., 2020), were also found overexpressed in PC9-Br (Figures 4B,D). Consistent with the resistance of PC9-Br to EGFR targeted TKIs, we found the expression of EGFR and p-EGFR were significantly reduced in PC9-Br compared to parental PC-9 (Figures 4C,D), which was regarded as an important mechanism of EGFR-TKI resistance in other EGFR-mutant NSCLC cell lines (Tabara et al., 2012; Nozaki et al., 2014; Xu et al., 2018). Similar regulations of these drug resistance related proteins were confirmed in animal derived BM cells PC9-BrM3 (Supplementary Figures 3A–D). What's more, NF-κB activation was reported to be responsible for the resistance of tumor cells to EGFR TKIs (Blakely et al., 2015; Lantermann et al., 2015; Li et al., 2020). Similarly, in our study, in addition to the decrease in EGFR/p-EGFR expression, we also found the significant activation of NF-κB pathway in BM cells compared to parental PC9 cells (Supplementary Figure 4). Collectively, these findings, on the one hand, revealed potential reasons for drug resistance occurred in both chip model derived PC9-Br and animal model derived PC9-BrM3 cells, which should be further investigated in future studies. One the other hand, consistent results obtained from both models suggested the chip platform is effective to recapitulate the pathologic changes during the course of *in vivo* metastasis and is an alternative model to further study the pathogenesis of brain metastasis.

## Conclusions

In summary, we established a model of lung cancer brain metastasis based on a newly microfluidic multi-organ chip and isolated the brain metastatic derivative cells (PC9-Br) of parental PC9 from the chip. Drug resistance to most chemotherapeutic agents and EGFR targeted TKIs were found to be developed in PC9-Br compared to parental PC9. Further proteomics revealed the different protein expression profile of PC9-Br and identified the hyperactive GSH metabolism pathway with the general overexpression of various GSH metabolism-related enzymes (GPX4, RRM2, GCLC, GPX1, GSTM4, GSTM1). Increased expression of aldehyde dehydrogenases (ALDH1A1, ALDH3A1), the well-known drug-resistance associated proteins, were also found in BM. What's more, the reduction of EGFR and phosphorylated EGFR expression, together with the significant activation of NF-κB pathway suggested that the PC9-Br cells had lost both the target of EGFR-TKIs and the addiction to EGFR signaling, which might result in the TKIs resistance in PC9-Br. In future studies, multi-omics analysis including genomics, proteomics and metabolomics will be conducted to further investigate novel mechanisms of the intrinsic drug resistance in BM.

## Data Availability Statement

The datasets presented in this study can be found in online repositories. The names of the repository/repositories and accession number(s) can be found below: http://www.proteomexchange.org/, PXD021760.

## Ethics Statement

The animal study was reviewed and approved by The Animal Ethics Review Committee of Dalian Medical University.

## Author Contributions

MX, YW, and WD designed and carried out the study. SX performed the statistical analysis. SW helped the fabrication of chips. WL wrote the first draft of the manuscript. MX and WD wrote sections of the manuscript. QW and WL conceived and supervised the project, designed and analyzed experiments, and revise the manuscript. All authors read and approved the submitted version.

## Conflict of Interest

The authors declare that the research was conducted in the absence of any commercial or financial relationships that could be construed as a potential conflict of interest.
