# Supplementary material for: Proteomic Reveals Reasons for Acquired Drug Resistance in Lung Cancer Derived Brain Metastasis Based on a Newly Established Multi-Organ Microfluidic Chip Model
Source: Front Bioeng Biotechnol. 2020 Dec 22;8:612091. doi: 10.3389/fbioe.2020.612091 (PMC7783320; doi:10.3389/fbioe.2020.612091)
Supplement: Supplementary file 1 [file Data_Sheet_1.docx]

Supplementary Material

**Materials and methods**

1. Protein Extraction

Cells was harvested and sonicated three times on ice using a high intensity ultrasonic processor (Scientz) in lysis buffer (8 M urea, 1% Protease Inhibitor Cocktail). The remaining debris was removed by centrifugation at 12,000 g at 4 °C for 10 min. Finally, the supernatant was collected and the protein concentration was determined with BCA kit according to the manufacturer’s instructions.

2. Trypsin Digestion

For digestion, the protein solution was reduced with 5 mM dithiothreitol for 30 min at 56 °C and alkylated with 11 mM iodoacetamide for 15 min at room temperature in darkness. The protein sample was then diluted by adding 100 mM TEAB to urea concentration less than 2M. Finally, trypsin was added at 1:50 trypsin-to-protein mass ratio for the first digestion overnight and 1:100 trypsin-to-protein mass ratio for a second 4 h-digestion.

3. TMT Labeling

After trypsin digestion, peptide was desalted by Strata X C18 SPE column (Phenomenex) and vacuum-dried. Peptide was reconstituted in 0.5 M TEAB and processed according to the manufacturer’s protocol for TMT kit. Briefly, one unit of TMT reagent were thawed and reconstituted in acetonitrile. The peptide mixtures were then incubated for 2 h at room temperature and pooled, desalted and dried by vacuum centrifugation.

4. HPLC Fractionation

The tryptic peptides were fractionated into fractions by high pH reverse-phase HPLC using Agilent 300Extend C18 column (5 μm particles, 4.6 mm ID, 250 mm length). Briefly, peptides were first separated with a gradient of 8% to 32% acetonitrile (pH 9.0) over 60 min into 60 fractions. Then, the peptides were combined into 18 fractions and dried by vacuum centrifuging.

5. LC-MS/MS Analysis

The tryptic peptides were dissolved in 0.1% formic acid (solvent A), directly loaded onto a home-made reversed-phase analytical column (15-cm length, 75 μm i.d.). The gradient was comprised of an increase from 6% to 23% solvent B (0.1% formic acid in 98% acetonitrile) over 26 min, 23% to 35% in 8 min and climbing to 80% in 3 min then holding at 80% for the last 3 min, all at a constant flow rate of 400 nL/min on an EASY-nLC 1000 UPLC system.

The peptides were subjected to NSI source followed by tandem mass spectrometry (MS/MS) in Q ExactiveTM Plus (Thermo) coupled online to the UPLC. The electrospray voltage applied was 2.0 kV. The m/z scan range was 350 to 1800 for full scan, and intact peptides were detected in the Orbitrap at a resolution of 70,000. Peptides were then selected for MS/MS using NCE setting as 28 and the fragments were detected in the Orbitrap at a resolution of 17,500. A data-dependent procedure that alternated between one MS scan followed by 20 MS/MS scans with 15.0s dynamic exclusion. Automatic gain control (AGC) was set at 5E4. Fixed first mass was set as 100 m/z.

6. Database Search

The secondary mass spectrometry data was retrieved using Maxquant (v1.5.2.8). Retrieval parameter settings: the database is SwissProt Human (20317 sequences), an anti-library is added to calculate the false positive rate (FDR) caused by random matching, and a common pollution library is added to the database to eliminate contaminated proteins in identification The effect of enzyme digestion is set to Trypsin / P; the number of missed cleavage sites is set to 2; the minimum length of peptides is set to 7 amino acid residues; the maximum number of modifications of peptides is set to 5; The ion mass error tolerance is set to 20 ppm and 5 ppm, respectively, and the mass error tolerance of the secondary fragment ion is 0.02 Da. The cysteine alkylation was set as a fixed modification, and the variable modification was the oxidation of methionine, the acetylation of the N-terminus of the protein, and the deamidation of asparagine and glutamine. The quantitative method was set to TMT-6plex, and the FDR for protein identification and PSM identification was set to 1%.

7. Bioinformatics Methods

The UniProt-GOA database (http://www.ebi.ac.uk/GOA/) gave Gene Ontology (GO) annotation. The KyotoEncyclopedia of Genes and Genomes (KEGG) database (https://www.genome.jp/kegg/tool/map_pathway2.html) identified enriched pathways. All identified differentially expressed proteins were entered into the KEGG database to search for related pathways. The output of the pathways was automatically classified into grade categories, and these grades are considered valid when the corrected p-value <0.05. DAVID (https://david.ncifcrf.gov/) served as a functional annotation tool to provide the enrichment results. The protein−protein interaction (PPI) network was created by STRING (https://string-db.org/) and PIPs (http://www.compbio.dundee.ac.uk/www-pips/). Then, import the text file with attributes into the open source software Cytoscape 3.5.2 (https://cytoscape.org/) for network visualization. For functional KEGG enrichment cluster analysis, the quantified proteins in this study were divided into four quantitative categories according to the quantification ratio firstly. Then, the quantitative category based enrichment and clustering were performed. For further hierarchical clustering based on different functional classification (such as: GO, Domain, Pathway, Complex). We first collated all the categories obtained after enrichment along with their p values, and then filtered for those categories which were at least enriched in one of the clusters with P value<0.05. This filtered P value matrix was transformed by the function x = −log10 (P value). Finally, these x values were z-transformed for each functional category. These z scores were then clustered by one-way hierarchical clustering (Euclidean distance, average linkage clustering) in Genesis. Cluster membership were visualized by a heat map using R-package “pheatmap”. According to the annotation of all the proteins identified, differentially expressed proteins (DEP) in each comparison group were performed enrichment analysis of GO, KEGG pathway and protein domain, respectively. A two-tailed Fisher’s exact test was employed to test the enrichment of the DEP against all identified proteins. The term with a corrected p value < 0.05 is considered as significant. In the bubble chart, the vertical axis is the functional category or pathway, and the horizontal axis value is the Log2 transformed ratio of DEP compared to identified proteins. The circle color indicates the significance p value, and the circle size indicates the number of differential proteins in terms.

**Figures**


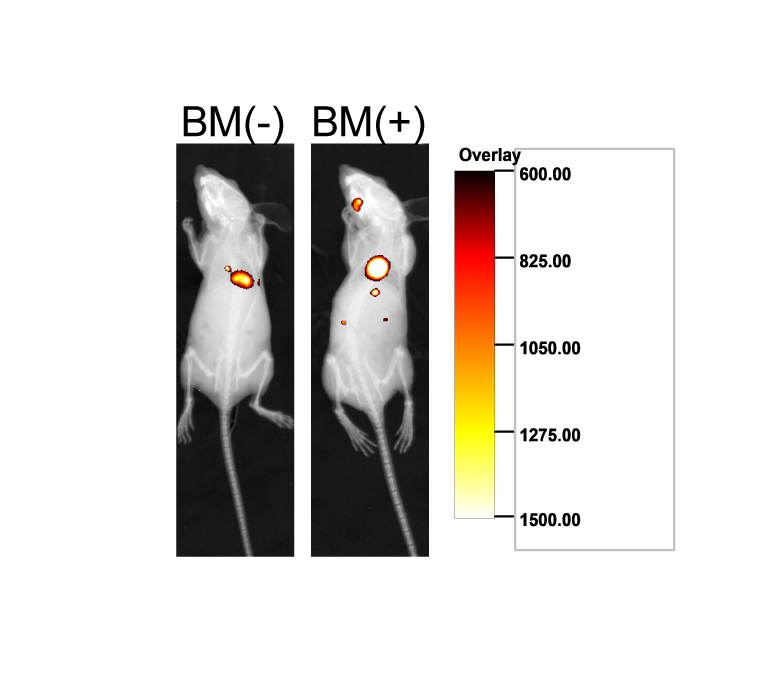


**Figure S1.** **Representative bioluminescent images of mice post intracardiac injection of tumor cells.** BM (-): No brain metastases. BM (+): Brain metastases positive.


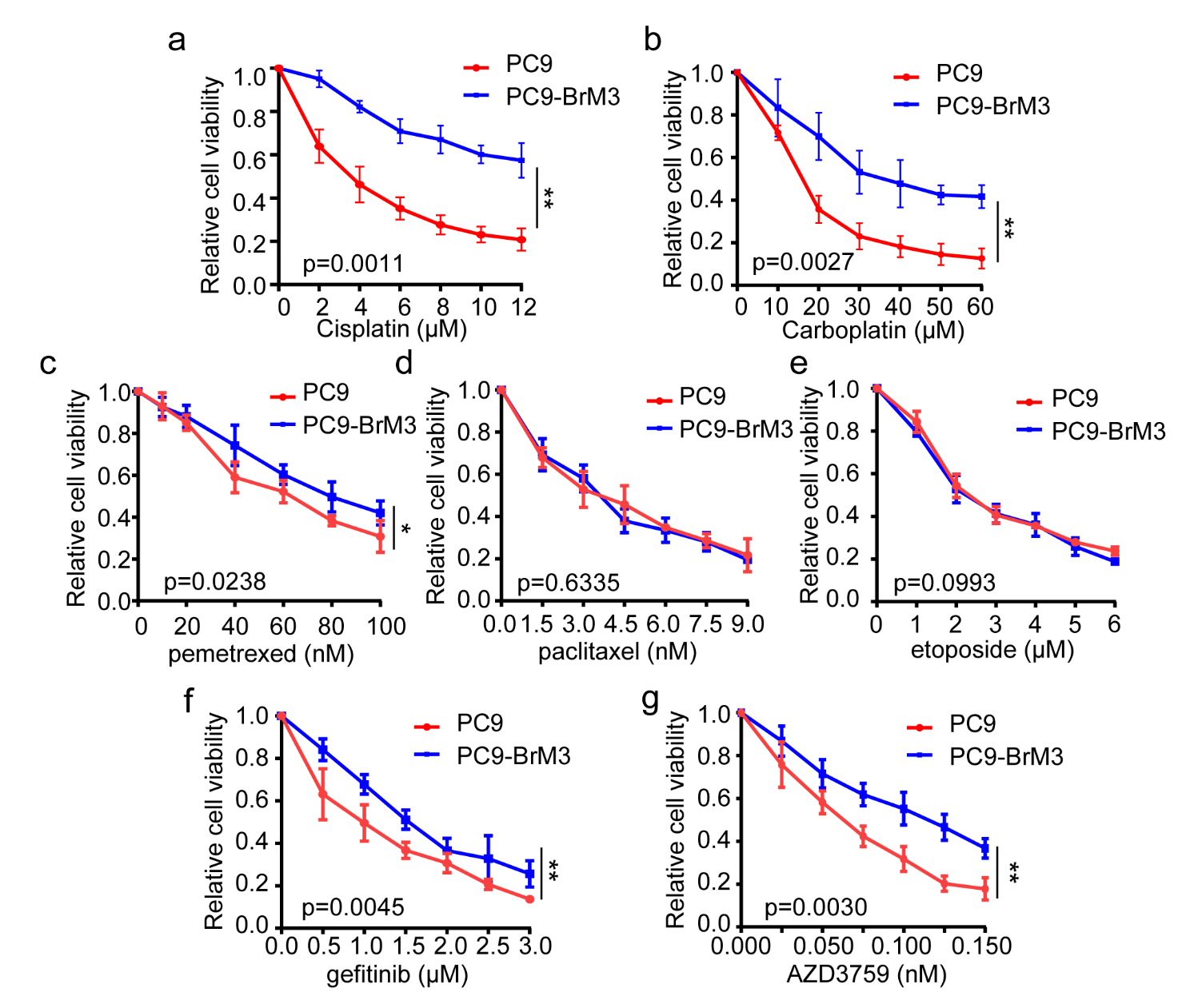


**Figure S2. The acquirement of obvious resistance to multiple anti-tumor drugs was found in PC9-BrM3 cells.** PC9 and PC9-BrM3 cells were treated with different doses of cisplatin (a), carboplatin (b), pemetrexed (c), paclitaxel (d), etoposide (e), gefitinib (f) and AZD3759 (g) for 72 h and CCK-8 assays were performed to determine their viability. n=3, *p<0.05, **p<0.01


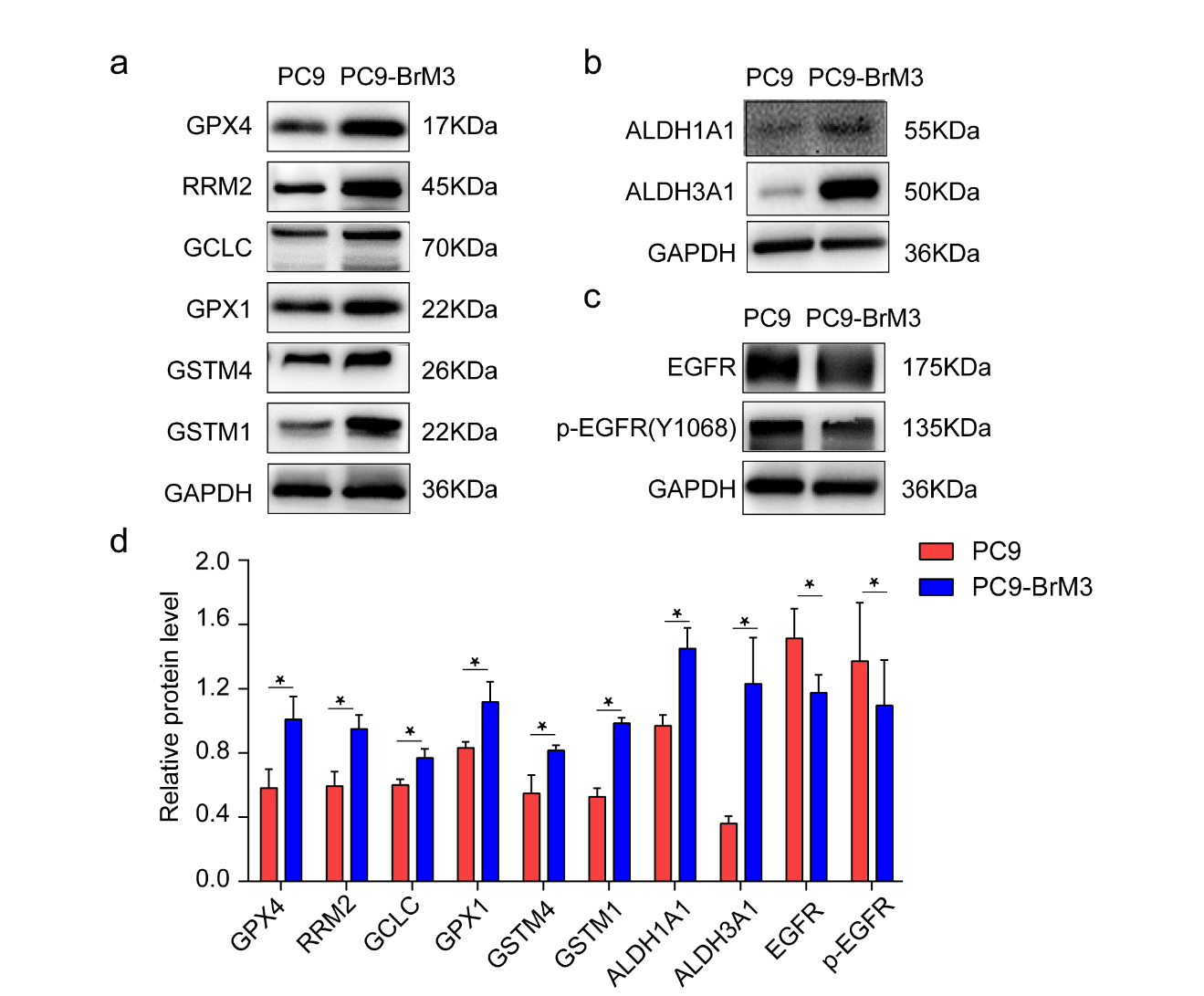


**Figure S3. Drug-resistance related proteins were confirmed to be regulated in PC9-BrM3 cells.** Representative western blot images showing the expression of GSH metabolism related enzymes (a), ALDH1A1 and ALDH3A1 (b), and EGFR/p-EGFR (c) in PC9 and PC9-BrM3 cells. (d) Quantitative results of western blotting images for proteins. n=3, *p<0.05


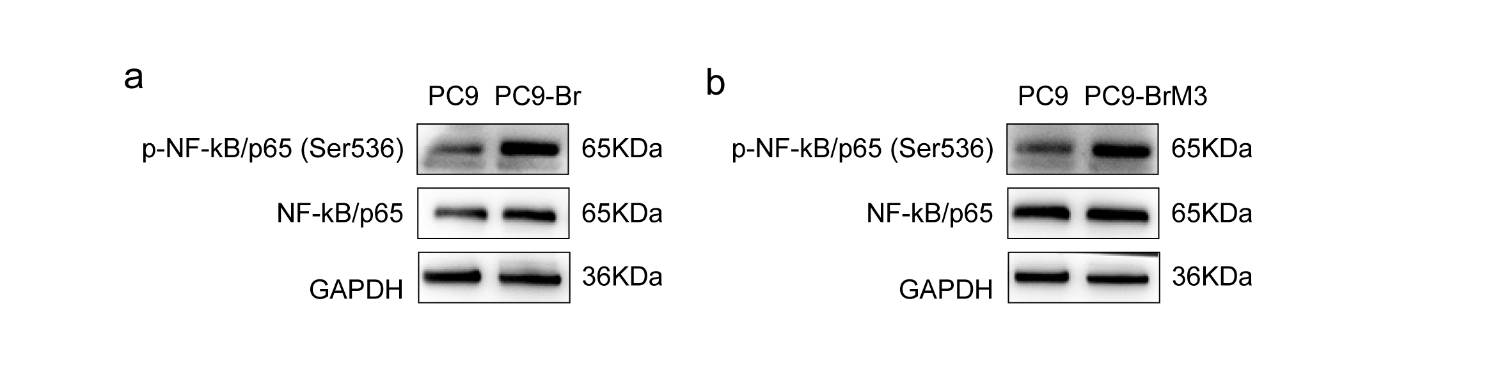


**Figure S4. NF-kB pathway was activated significantly in BM.** Representative western blot images showing the activation of NF-kB pathway in PC9-Br (a) and PC9-BrM3 (b) cells.
